# Sensor for High Speed, High Precision Measurement of 2-D Positions

**DOI:** 10.3390/s91108810

**Published:** 2009-11-03

**Authors:** Carlos A. Luna, José L. Lázaro, Manuel Mazo, Angel Cano

**Affiliations:** Electronics Department, High Polytechnic School, Alcalá University, Alcalá de Henares (28871), Madrid, Spain; E-Mails: mazo@depeca.uah.es (M.M); lazaro@depeca.uah.es (J.L.L.); angel_cano_78@yahoo.es (A.C)

**Keywords:** line-scan calibration, object detection, 2-D measurements, computer vision, object position

## Abstract

A sensor system to measure the 2-D position of an object that intercepts a plane in space is presented in this paper. This sensor system was developed with the aim of measuring the height and lateral position of contact wires supplying power to electric locomotives. The sensor comprises two line-scans focused on the zone to be measured and positioned in such a way that their viewing planes are on the same plane. The report includes a mathematical model of the sensor system, and details the method used for calibrating the sensor system. The procedure used for high speed measurement of object position in space is also described, where measurement acquisition time was less than 0.7 ms. Finally, position measurement results verifying system performance in real time are given.

## 1. Introduction

The use of visual information to detect the position of objects in relation to other objects is a fundamental function of computer vision systems. Many methods and applications have been developed in order to perform this task, all with their respective advantages and disadvantages with regards to computational efficiency, complexity, robustness, accuracy and performance. In the majority of cases, more than one camera [1-4], or a camera and a structured light source [5-7] have been used in order to establish the position of an object. In some applications, the use of line-scans has contributed to an overall improvement of the system, as measurement acquisition is faster than with matrix cameras, less information needs to be processed, and sensors with greater spatial resolution (higher number of pixels) can be used [8,9]. However, a disadvantage of linear sensors is that neither traditional calibration methods nor the object detection algorithms developed for matrix camera based systems can be applied [10-12]. The calibration patterns used in calibration of the matrix cameras, used in 3D measurements, can not be used for calibration of line-scan sensors, because it is virtually impossible to match the captured line-image with the interest points of the patterns (circles centers, interception lines).

In the calibration of line-scan sensors, we cannot get 3D measurements without *a priori* assumption of one coordinate, so it is more accurate in this case to optimize the parameters for a 2D calibration. A line-scan calibration method using calibration pattern line-images in different positions is presented in [13]. Although this requires extreme precision when positioning the pattern, and could thus represent a disadvantage, [14] describes the resolution of this potential problem through the use of calibration patterns at different depths. Nevertheless, when more than one line scan is used, these methods are only capable of obtaining individual intrinsic parameters for each line-scan. However, it is not possible to obtain accurate extrinsic parameters, as one of the limitations of these methods is that the line-scan sensor array must be approximately parallel to the calibration pattern planes. If two line-scans are used to measure position with triangulation techniques, both sensors must be separated and thus the pattern cannot be situated in such as way that the planes are parallel to the sensor arrays.

In this study, we used a 2-D sensor based on two line-scans. Section 2 describes the sensor employed and the sensor modeling. Section 3 presents the calibration method used. Section 4 gives the experimental results. Finally, Section 5 summarizes the main conclusions.

## 2. Sensor System

The overall function of the sensor system is based on the capture of contact wire images with two line-scans. Following line-image processing and triangulation, it is possible to calculate 2-D coordinates for the objects in relation to a specific reference system.

**Figure 1.**
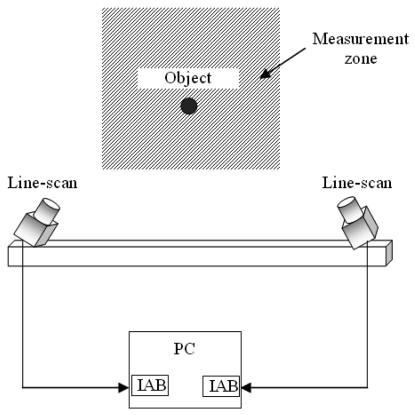
Block diagram of 2-D sensor system.

The system comprises a computer with an image acquisition and processing board (IAB) for each line-scan, as shown in Figure 1. This board is responsible for information transfer (images and control) between the cameras and the PC. The PC performs line-image processing to determine the 2-D coordinates for objects. Image acquisition, control, processing and data presentation is carried out using software in C language.

### 2.1. Sensor Modeling

**Figure 2.**
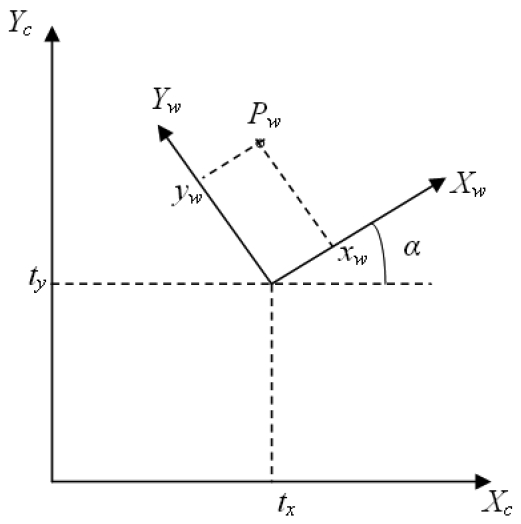
Relation between line-scan coordinate system and world coordinate system.

The reference coordinate system does not usually coincide with camera or line-scan coordinates (Figure 2). To resolve this issue, and thus obtain coordinates for the line-image of a 2-D point in space with respect to a reference system, a projective transformation in 2-D is performed. This enabled us to obtain camera system coordinates which corresponded to a scene point.

Using Figure 2 and a 2-D projective transformation, it was possible to change from one coordinate system to the other (Equation 1):

(1) $${\text{P}}_{c}=\text{R}\cdot {\text{P}}_{w}+\text{T}$$

where **R** is the rotation matrix defined by:

(2) $$\text{R}=\left[\begin{array}{cc} r_{11} & r_{12}\\ r_{21} & r_{22} \end{array}\right]=\left[\begin{array}{cc} \cos(\alpha ) & -\sin(\alpha )\\ \sin(\alpha ) & \cos(\alpha ) \end{array}\right]$$

and **T** is the translation vector which defines the relative position between the optical centre of the line-scan camera and the world coordinates centre (Equation 3):

(3) $$\text{T}=\left[\begin{array}{c} t_{x}\\ t_{y} \end{array}\right]$$

If Equation (1) is expressed by homogeneous coordinates, we obtain Equation (4):

(4) $$\left[\begin{array}{c} x_{C}\\ y_{C}\\ 1 \end{array}\right]=\left[\begin{array}{ccc} r_{11} & r_{12} & t_{X}\\ r_{21} & r_{22} & t_{Y}\\ 0 & 0 & 1 \end{array}\right]\cdot \left[\begin{array}{c} x_{W}\\ y_{W}\\ 1 \end{array}\right]$$

the values represented in the projection model were calculated through camera calibration. These extrinsic parameters (*t_x_, t_y_, α*) link the relative position between the world coordinate system and the camera coordinate system.

Using the pin-hole camera model for a 1-D sensor, as is the case of line-scans, the projection of a point **P***_c_*(*x_c_, y_c_*) from the scene onto a line-image will bear the coordinate *x*:

(5) $$x=f\cdot \frac{x_{C}}{y_{C}}$$

If (5) is shown in matrix form and with homogeneous coordinates, we obtain:

(6) $$\left[\begin{array}{c} m\cdot x\\ m \end{array}\right]=\left[\begin{array}{ccc} f & 0 & 0\\ 0 & 1 & 0 \end{array}\right]\cdot \left[\begin{array}{c} x_{c}\\ y_{c}\\ 1 \end{array}\right]$$

Substituting (4) in (6), a general expression is obtained for relating a point in the world coordinate system with its corresponding projection onto the line-image:

(7) $$\left[\begin{array}{c} m\cdot x\\ m \end{array}\right]=\left[\begin{array}{ccc} f & 0 & 0\\ 0 & 1 & 0 \end{array}\right]\cdot \left[\begin{array}{ccc} r_{11} & r_{12} & t_{x}\\ r_{21} & r_{22} & t_{y}\\ 0 & 0 & 1 \end{array}\right]\cdot \left[\begin{array}{c} x_{w}\\ y_{w}\\ 1 \end{array}\right]$$

**Figure 3.**
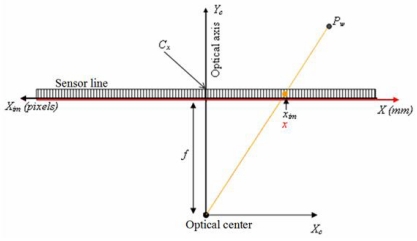
Diagram explaining the pin-hole model for a line-scan.

A diagram explaining the pin-hole model for a line-scan is shown In Figure 3. If *x* is represented by pixel coordinates *x_im_*, and we take into account that the optical axis may coincide with a pixel *c_x_* different to the centre of the sensor, it is then possible to formulate the Equation (8).

(8) $$x_{im}=-\frac{x}{s_{X}}+c_{X}$$

The scale factor *s_x_* (*mm/pixel*) is the parameter which relates the line-image system of metric coordinates to the pixel array coordinate system provided by the line-scan. This value corresponds to pixel size. In this case, the theoretical value given by the manufacturer is 12 μm.

Substituting (5) in (8) and doing *fx* = *f/s_x_*, the Equation (9) is obtained, which models the line-scans according to the parameters of the pin-hole model:

(9) $$x_{im}=-f_{X}\cdot \frac{x_{C}}{y_{C}}+c_{X}$$

Using (9), Equation (7) can be rewritten in the following form:

(10) $$\left[\begin{array}{c} m\cdot x_{im}\\ m \end{array}\right]=\underset{{\text{M}}_{\mathrm{int}}}{\underset{︸}{\left[\begin{array}{cc} -f_{x} & c_{x}\\ 0 & 1 \end{array}\right]}}\cdot \underset{{\text{M}}_{\text{ext}}}{\underset{︸}{\left[\begin{array}{ccc} r_{11} & r_{12} & t_{x}\\ r_{21} & r_{22} & t_{y} \end{array}\right]}}\cdot \left[\begin{array}{c} x_{w}\\ y_{w}\\ 1 \end{array}\right]={\text{M}}_{\mathrm{int}}\cdot {\text{M}}_{\text{ext}}\cdot \left[\begin{array}{c} x_{w}\\ y_{w}\\ 1 \end{array}\right]$$

When the intrinsic parameter matrix **M***_int_* and the extrinsic parameter matrix **M***_ext_* are multiplied, a general expression for the projection matrix **M = M***_int_* · **M***_ext_* is obtained, which represents the relation between the scene points and their projection onto a line-image.

If the matrix coefficients are represented by *m_11_*…*m_23_*, (10) they can be rewritten as (11):

(11) $$\left[\begin{array}{c} m\cdot x_{im}\\ m \end{array}\right]=\underset{\text{M}}{\underset{︸}{\left[\begin{array}{ccc} m_{11} & m_{12} & m_{13}\\ m_{21} & m_{22} & m_{23} \end{array}\right]}}\cdot \left[\begin{array}{c} x_{w}\\ y_{w}\\ 1 \end{array}\right]$$

## 3. Calculation of Calibration Parameters

An alternative method for obtaining projection matrix **M** coefficients is to assign a value to one of the coefficients (in this case, the value *m_23_* = 1 is chosen), and to express the other projection matrix coefficients according to this value (12). Thus, the Direct Linear Transformation (DLT) coefficient vector **L***^T^* = [*L_1_ L_2_ L_3_ L_4_ L_5_*] is obtained:

(12) $$\left[\begin{array}{ccc} \overset{m_{11}}{\;}/\underset{m_{23}}{\;} & \overset{m_{12}}{\;}/\underset{m_{23}}{\;} & \overset{m_{13}}{\;}/\underset{m_{23}}{\;}\\ \overset{m_{21}}{\;}/\underset{m_{23}}{\;} & \overset{m_{22}}{\;}/\underset{m_{23}}{\;} & 1 \end{array}\right]=\left[\begin{array}{ccc} L_{1} & L_{2} & L_{3}\\ L_{4} & L_{5} & 1 \end{array}\right]$$

If the DLT coefficient vector is substituted, and the matrices in (11) are multiplied, the unknown quantity producing scene point projection onto the line-image is found:

(13) $$x_{im}=\left[x_{w}\;y_{w}\;1\;-x_{w}\cdot x_{im}\;-y_{w}\cdot x_{im}\right]\cdot {\text{L}}^{T}$$

As can be seen, Equation (13) has five DLT coefficients (*L_1_* … *L_5_*), so at least five 2-D point correspondences, visible to both line-scans, are necessary. Therefore, the pattern must have at least five known points to establish their correspondence with the captured line-image.

The number of points of correspondence between the real world and line-images is represented by *h*. The more points used, the greater calibration accuracy becomes. A matrix of *h* rows is formed, where each row corresponds to a point in the pattern:

(14) $$\underset{\text{B}}{\underset{︸}{\left[\begin{array}{c} x_{im1}\\ \cdot \\ \cdot \\ x_{\text{imh}} \end{array}\right]}}=\underset{\text{A}}{\underset{︸}{\left[\begin{array}{ccccc} x_{w1} & y_{w1} & 1 & -x_{w1}\cdot x_{im1} & -y_{w1}\cdot x_{im1}\\ \cdot & \cdot & \cdot & \cdot & \cdot \\ \cdot & \cdot & \cdot & \cdot & \cdot \\ x_{wh} & y_{wh} & 1 & -x_{wh}\cdot x_{\text{imh}} & -y_{wh}\cdot x_{\text{imh}} \end{array}\right]}}\cdot {\text{L}}^{T}$$

To find **L**, the least squares estimate is used:

(15) $$\text{L}=\text{A}{\left({\text{A}}^{T}\cdot \text{A}\right)}^{-1}\cdot {\text{A}}^{T}\cdot \text{B}$$

The use of *m_23_* = 1 is justified because the solution is subject to a scale factor, given that the projection matrix is homogeneous. The parameter *m_23_* is the *t_y_* component of the translation vector which locates the line-scan in the world 2-D reference system. Thus, if *t_y_* were null, it would not be valid for the proposed solution. The parameter *m_23_* is obtained from the *L_4_* and *L_5_* parameters of the DLT coefficient matrix:

(16) $$m_{23}=\frac{1}{\sqrt{L_{4}^{2}+L_{5}^{2}}}=t_{y}$$

To obtain the projection matrix **M** from the DLT coefficients, an inverse scale change is carried out. This is achieved by multiplying each of the elements calculated by *m_23_*:

(17) $$\text{M}=m_{23}\;\left[\begin{array}{ccc} L_{1} & L_{2} & L_{3}\\ L_{4} & L_{5} & 1 \end{array}\right]$$

Intrinsic parameters

Once the projection matrix has been calculated, calculation of the intrinsic parameters is a simple operation:

(18) $$\left[\begin{array}{c} -f_{x}\\ c_{x} \end{array}\right]={\left[\begin{array}{cc} m_{23}\cdot L_{5} & m_{23}\cdot L_{4}\\ -m_{23}\cdot L_{4} & m_{23}\cdot L_{5} \end{array}\right]}^{-1}\cdot \left[\begin{array}{c} m_{23}\cdot L_{1}\\ m_{23}\cdot L_{2} \end{array}\right]$$

Extrinsic parameters

The extrinsic parameters are obtained as follows:

(19) $$t_{y}=m_{23}=\frac{1}{\sqrt{L_{4}^{2}+L_{5}^{2}}}$$

(20) $$\alpha =\sin^{-1}\left(m_{23}\cdot L_{5}\right)$$

(21) $$t_{x}=\frac{c_{x}\cdot t_{y}-m_{23}\cdot L_{2}}{f_{x}}$$

### 3.1. Calibration Pattern

**Figure 4.**
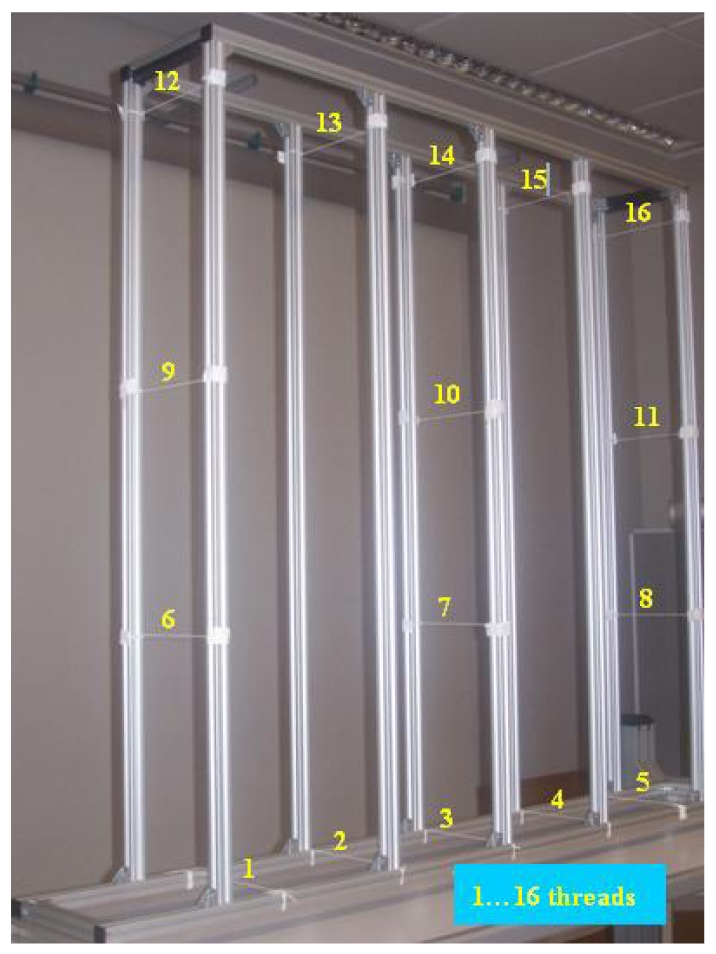
Calibration pattern comprising threads.

**Figure 5.**
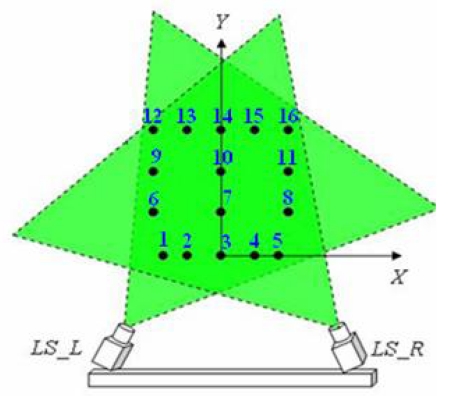
Diagram that show the viewing planes crossed by calibration pattern threads.

A fundamental steep in the calibration process is the selection of an adequate calibration pattern. As for the calibration of matrix cameras, 3-D patterns offer the best results for line-scans calibration. In this case, calibration was carried out using a 3-D calibration pattern, comprising a series of parallel threads in different positions (Figure 4). The pattern is located in such a way that the threads cross the vision plane of the line-scans perpendicularly, as shown in Figure 5.

When many reference threads are used in the proposed pattern, overlapping of the different threads projected may occur. This can be detected by the lack of concordance between the number of reference points in our pattern and the number of points seen in the line-images.

### 3.2. Calibration Results

In our case, calibration was carried out using a total of 16 threads in the pattern. Table 1 gives the calibration parameters obtained, and calibration error, *ε*. This error quantifies the difference between the coordinates for each point on the real line-images *x_im_real_* with respect to those calculated by means of its projection *x_im_proy_*. Applying the projection matrix *x_im_* for each of the calibration pattern *h* points:

(22) $$\varepsilon =\frac{1}{h}\sum_{i=1}^{h}\left(x_{\text{im\_proy}\;i}-x_{\text{im\_real}\;i}\right)$$

With the value of *s_x_* = 12 μm and the scale factors obtained, focal length for each line-scan can be calculated. Focal length of the left hand line-scan is *f_L_* = 30.7487 mm, and focal length of the right hand line-scan is *f_R_* = 30.4995 mm.

### 3.3. Calculation of 2-D Position with Two Calibrated Line-scan

Once both line-scans have been calibrated, it is possible to obtain a correspondence between a 2-D point and its projection on both line-images. The model for a single calibrated line-scan, based on a pin-hole model, can be expressed by (11). If the same calibration pattern, located in a particular position, is used to calibrate both line-scans, the Equation (23) can be obtained, which establishes the relation between the two previous models, and yields the parameters [*x_w_,y_w_*] according to the line-images captured for each line-scan, and the corresponding projection matrices:

(23) $$\begin{array}{c} \left[\begin{array}{ccc} m_{11}^{R} & m_{12}^{R} & -x_{im}^{R}\\ m_{21}^{R} & m_{22}^{R} & -1 \end{array}\right]\cdot \left[\begin{array}{c} x_{w}\\ y_{w}\\ m^{R} \end{array}\right]=\left[\begin{array}{c} -m_{13}^{R}\\ -m_{23}^{R} \end{array}\right]\\ \left[\begin{array}{ccc} m_{11}^{L} & m_{12}^{L} & -x_{im}^{L}\\ m_{21}^{L} & m_{22}^{L} & -1 \end{array}\right]\cdot \left[\begin{array}{c} x_{w}\\ y_{w}\\ m^{L} \end{array}\right]=\left[\begin{array}{c} -m_{13}^{L}\\ -m_{23}^{L} \end{array}\right] \end{array}\}$$

the system of linear equations (23) represents two straight lines which are cut at the points [*x_w_,y_w_*]. In this system of equations, the other unknown parameters are *m^L^* and *m^R^*. To obtain the geometric location of the points [*x_w_,y_w_*], an inverse operation to that carried out for Equation (23) is performed, giving (24):

(24) $$\left[\begin{array}{c} x_{w}\\ y_{w}\\ m^{L}\\ m^{R} \end{array}\right]={\left[\begin{array}{cccc} m_{11}^{L} & m_{12}^{L} & -x_{im}^{L} & 0\\ m_{21}^{L} & m_{22}^{L} & -1 & 0\\ m_{11}^{R} & m_{12}^{R} & 0 & -x_{im}^{R}\\ m_{21}^{R} & m_{22}^{R} & 0 & -1 \end{array}\right]}^{-1}\cdot \left[\begin{array}{c} -m_{13}^{L}\\ -m_{23}^{L}\\ -m_{13}^{R}\\ -m_{23}^{R} \end{array}\right]$$

with the projection matrices for each line-scan and the Equation (24), it is possible to calculate the 2-D position of a point in the measurement zone, whose projection in each line-image is $x_{im}^{L}$ and $x_{im}^{R}$. Using the *n* (*n* = 2048) values of $x_{im}^{L}$ and $x_{im}^{R}$, we calculated two matrices of *n*x*n* (2048 × 2048), where each value corresponded to the position in *x_w_* (lateral decentring) and the position in *y_w_* (height). We called these matrices the “Sensor Matrices”.

To summarise, the system for measuring 2-D position is defined by two matrices, called “sensor matrices”, which contain the coordinates (*x,y*) for each scene point projected onto the line-scans at the coordinate $x_{im}^{L}$ and $x_{im}^{R}$. By reading these matrices, the geometric location of a scene point can be identified. For this, it is only necessary to know pixel position in the projection of the object to be measured on each line-scan. These pixel values are then used to read the sensor matrices, stored during the calibration process. In this way, the calculation time for measurements carried out in real time is reduced.

## 4. Experimental Results

In this section, we describe some practical experiments which were carried out with the aim of verifying the performance of the measuring system proposed. The experiments were aimed at establishing the accuracy of measurements under real operating conditions. In order to achieve this, measurements were taken of a moving contact wire, to verify efficiency of the tracking algorithms. In addition, static measurements were taken of the threads in different positions, in order to calculate magnitude of error in measurements.

### 4.1. Measuring the 2-D Position of Static Objects

**Figure 6.**
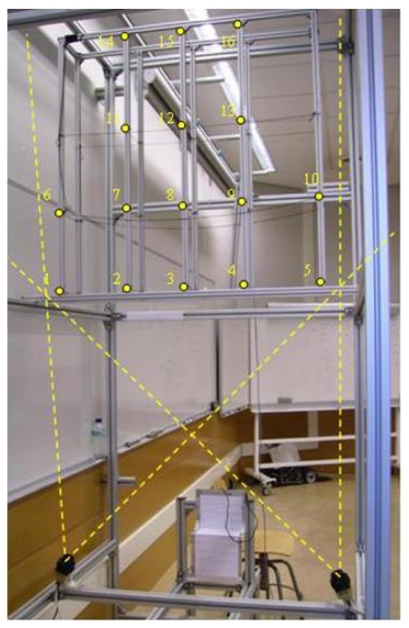
Diagram showing the planes of vision to be crossed by the calibration pattern threads. The distance between the line-scans is 106.5 cm, and the angles *α_LS_L_* = 67.68 degrees and *α_LS_R_* = 67.28 degrees.

The aim of this experiment was to verify the accuracy of lateral decentring (*X*) and height (*Y*) measurements taken with the system when the system sensor and the measured objects were static. The calibration pattern structure, with 16 white 0.5 mm diameter threads, was placed in a known position with the coordinates (*X_S1real_,Y_S1real_*). The base reference system was situated at a central point between the two line-scans. Once the position measurements had been taken with the system sensor (*X_S1sensor_,Y_S1sensorl_*), the values obtained were compared with the real values. Figure 6 shows the calibration pattern with the 16 reference threads placed in the sensor measurement zone. The various threads are numbered and marked by a yellow dot.

As an example, Table 2 gives both real and measured coordinate values for the threads in one particular calibration pattern position. The same experiment was carried out for different thread and pattern positions, but always ensuring that the position of the threads within the measurement zone coincided. From a total of 524 measurements, maximum error of *x* was 2.1 mm, and standard error was 0.82 mm. For height measurements (*y*), maximum error was 3.2 mm and standard error was 0.94 mm. This error was due to incorrect thread placement, calibration error and/or sensor system resolution.

### 4.1. Measuring the 2-D Position of a Moving Object

This second experiment aimed to verify the validity of the monitoring algorithm and the system's capacity for measuring the position of an object (contact wire) moving at high speed.

**Figure 7.**
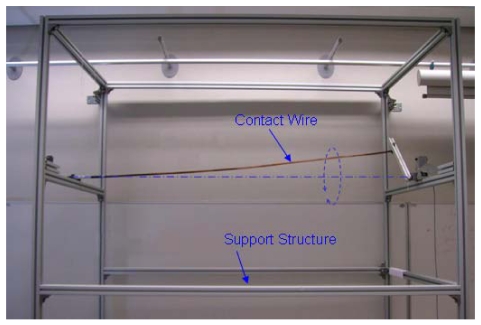
Structure assembled in order to generate contact wire movement.

To move the contact wire one end was attached to a bearing placed in a constant position so that only the wire could rotate. The other end was attached to another support located on an aluminium bar which in turn was attached to an engine rotor. The engine rotates the aluminium bar parallel to the plane of view of the line-scans. The rotor axis was positioned so that whatever the position of the rotating bar, the contact wire was always located within the field of vision of the cameras. A photograph of the experimental structure assembled in order to generate contact wire movement is shown in Figure 7.

**Figure 8.**
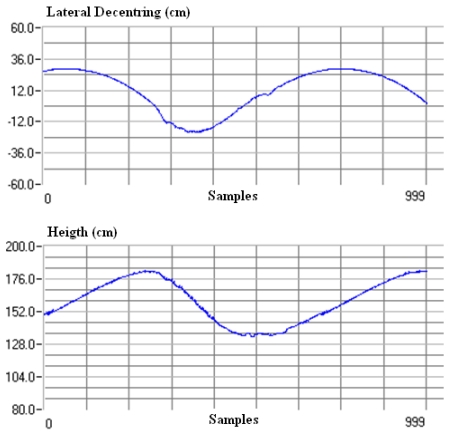
Contact wire measurements at a sample speed of 100 fps: Height and lateral decentring.

The graphs in Figure 8 give height and decentring measurements at a sample speed of 100 frames per second (fps). The small jumps in the curves are due to the experimental structure used.

To verify real time sensor system operation, line-scan tests were carried out for various acquisition times. Maximum acquisition and processing speed without loss of samples was 1,430 frames per second, using a PC with P4 2.0 GHz processor and 512 MB of RAM. This high speed was achieved because it was only necessary to find centroids of the line-images captured. Once the centroids had been obtained, a matrix reading is sufficient to obtain the values of *x* and *y*.

## 5. Conclusions

This paper has presented a 2-D sensor system based on two line-scans. Among other applications, it can be used to verify the geometry of contact wires supplying power to electric locomotives. A mathematical model has been reported.

In addition, has been proposed and described a method for calibrating the sensor system. This method is based on the calibration of both line-scans using the calibration pattern position. The calibration pattern is a 3-D structure with various parallel threads attached. Calibration provides the matrices containing the coordinates (*x,y*) for each scene point, corresponding to the projection of these points onto each line-scan. To obtain the coordinates (*x,y*) is only necessary to know pixel position in the projection of the object in each line-scan. These pixel values are then used to read the sensor matrices which contain the coordinates (*x,y*). In this way, processing time may be less than 0.7 ms.

Experiments were carried out in order to verify system operation. The first experiment examined static measurement error, and from a total of 524 measurements, the maximum error of *x* was found to be 2.1 mm, with a standard error of 0.82 mm. In the case of height measurement (*y*), maximum error was 3.2 mm and standard error was 0.94 mm. The second experiment measured contact wire position when moving, in order to verify monitoring algorithms. Maximum acquisition and processing speed without sample loss was 1430 frames per second, using a PC with P4 2.0 GHz processor and 512 MB of RAM.

## Figures and Tables

**Table 1.** Calibration results.

**Parameter**	**Left hand Line-scan**	**Right hand Line-scan**
*t_x_*, cm	53.34	−53.35
*t_y_*, cm	106.35	106.12
*α*, degrees	−21.8	21.7
*f_x_*	2,577.0	2,565.9
*c_x_*, pixels	1,033.9	1,019.7
ε, pixels	0.63	0.49

**Table 2.** Real and sensor measured coordinates for different points (threads).

**Thread**	***X*_*S*1 real_** **mm**	***X*_*S*1 sensor_** **mm**	**Error** **mm**	***Y*_*S*1 real_** **mm**	***Y*_*S*1 sensor_** **mm**	**Error** **mm**
1	−490	−489.6322	0.3678	1,070	1,069.5631	0.4369
2	−220	−219.7668	0.2332	1,070	1,071.817	1.817
3	0	0.1267	0.1267	1,070	1,069.8788	0.1212
4	220	221.2802	1.2802	1,070	1,069.5836	0.4164
5	490	489.8626	0.1374	1,070	1,071.0224	1.0224
6	−490	−490.0087	0.0087	1,370	1,368.6644	1.3356
7	−220	−220.5975	0.5975	1,370	1,369.9805	0.0195
8	0	−0.5668	0.5668	1,370	1,369.8184	0.1816
9	220	219.1745	0.8255	1,370	1,370.5471	0.5471
10	490	490.9952	0.9952	1,370	1,369.9767	0.0233
11	−220	−220.2104	0.2104	1,670	1,671.0066	1.0066
12	0	−0.4616	0.4616	1,670	1,669.8844	0.1156
13	220	219.5798	0.4202	1,670	1,668.9608	1.0392
14	−220	−219.9084	0.0916	2,030	2,031.5914	1.5914
15	0	1.7755	1.7755	2,030	2,029.9706	0.0294
16	220	218.9772	1.0228	2,030	2,030.5424	0.5424
